# Supplementary material for: RMR-Related MAP2K6 Gene Variation on the Risk of Overweight/Obesity in Children: A 3-Year Panel Study
Source: J Pers Med. 2021 Feb 2;11(2):91. doi: 10.3390/jpm11020091 (PMC7913067; doi:10.3390/jpm11020091)

Supplementary Table 1.

The primer sequences of seven *MEK6* SNPs.

rs number	Strand	Primer sequence		Tm	Additive
rs12603937	Forward	Forward	CTTTTTCCAGGTGAAGGAC	55	Betaine
		Reverse	tattggaacacagccacac		
		Genotyping	GTGTATTSTTTAAGATCATGTCATTT		
rs11654541	Forward	Forward	GCCTCCTTTCTGATGTGAT	55	Betaine
		Reverse	CCCTGTTAAGGAGTTTCCA		
		Genotyping	CTTTGCCATACAGAATGCTATAAAAA		
rs10512573	Reverse	Forward	TGAATTTCAGAGCCTTCAA	55	Betaine
		Reverse	CTAGCAAGTGCCTTCATCA		
		Genotyping	tgaaaagattaccttccactaaag		
rs2285601	Forward	Forward	AGCATGCCTTTGTTTTCTT	55	Betaine
		Reverse	TCCCCCATCAATGTTATTT		
		Genotyping	GGTGTTAACATAGGTTAAGTTTTG		
rs2285600	Forward	Forward	ACCTTTTCATTTCCCTCCT	55	Betaine
		Reverse	TTCCTTTCTCCTCAGCAGT		
		Genotyping	CATATTTTTAAAAAGTAGGTTTCTGAT		
rs9916229	Reverse	Forward	ttggttttgtgtaaagagca	55	Betaine
		Reverse	TCTAACACCACCAGAAGCA		
		Genotyping	tgttgaactccagattcttctctg		
rs756942	Reverse	Forward	AATGCTGTTTTAGGGATGC	55	Betaine
		Reverse	AGAACAACTCTTCGCCTTC		
		Genotyping	caataccagaaatttcctgaatttgga		

Supplementary Table 2.

The relative frequencies (RF) of obesity including overweight (%), in wild and mutant allele each of 7 SNPs in total, boys and girls.

Gene SNPs	Relative Frequency Major	Minor	BMI		Allele wild vs mutant	OB Fre		OB Fre	Chi-Sq (P-value)
rs 12603937	0.746	0.254			G	OB Fre	T	OB Fre	
			Total	Non-OB	288(58.5%)	21.9%	80(16.3%)	34.5%	8.281
				OB	81(16.5%)		43(8.7%)		(0.004)
			Boys	Non-OB	212(60.2%)	18.7%	66(18.8%)	27.5%	3.075
				OB	49(13.9%)		25(7.1%)		(0.08)
			Girls	Non-OB	76(54.3%)	29.6%	14(10%)	56.3%	7.619
				OB	32(22.9%)		18(12.9%)		(0.006)
rs 11654541	0.738	0.262			G		T		
			Total	Non-OB	283(57.5%)	22.3%	83(16.9%)	35.2%	8.276
				OB	81(16.5%)		45(9.1%)		(0.004)
			Boys	Non-OB	207(58.8%)	19.2%	69(19.6%)	28.2%	3.329
				OB	49(13.9%)		27(7.7%)		(0.068)
			Girls	Non-OB	76(54.3%)	29.6%	14(10%)	56.3%	7.619
				OB	32(22.9%)		18(12.9%)		(0.006)
rs 1051273	0.739	0.261			G		A		
			Total	Non-OB	285(57.7%)	21.9%	83(16.8%)	34.5%	8.468
				OB	81(16.4%)		45(9.1%)		(0.004)
			Boys	Non-OB	209(59%)	18.9%	69(19.5%)	28.2%	3.461
				OB	49(13.8%)		27(7.6%)		(0.063)
			Girls	Non-OB	76(54.3%)	29.6%	14(10%)	56.3%	7.619
				OB	32(22.9%)		18(12.9%)		(0.006)
rs 2285601	0.742	0.258			T		A		
			Total	Non-OB	280(58.1%)	21.8%	80(16.6%)	35.5%	9.139
				OB	78(16.2%)		44(9.1%)		(0.003)
			Boys	Non-OB	206(59.2%)	18.6%	68(19.5%)	28.4%	3.997
				OB	47(13.5%)		27(7.8%)		(0.056)
			Girls	Non-OB	74(55.2%)	29.5%	12(9%)	58.6%	8.368
				OB	31(23.1%)		17(12.7%)		(0.004)
rs 2285600	0.741	0.259			G		A		
			Total	Non-OB	286(57.9%)	22.1%	82(16.6%)	35.4%	8.866
				OB	81(16.4%)		45(9.1%)		(0.003)
			Boys	Non-OB	210(59.3%)	18.9%	68(19.2%)	28.4%	3.722
				OB	49(13.8%)		27(7.6%)		(0.058)
			Girls	Non-OB	76(54.3%)	29.6%	14(10%)	56.3%	7.619
				OB	32(22.9%)		18(12.9%)		(0.006)
rs 9916229	0.675	0.325			C		A		
			Total	Non-OB	258(52.2%)	21.6%	110(22.3%)	33.3%	7.989
				OB	71(14.4%)		55(11.1%)		(0.005)
			Boys	Non-OB	193(54.5%)	18.2%	85(24%)	27.9%	4.432
				OB	43(12.1%)		33(9.3%)		(0.035)
			Girls	Non-OB	65(46.4%)	30.1%	25(17.9%)	46.8%	3.793
				OB	28(20%)		22(15.7%)		(0.059)
rs 756942	0.395	0.305			C		T		
			Total	Non-OB	267(54%)	21.7%	101(20.4%)	33.9%	8.39
				OB	74(15%)		52(10.5%)		(0.004)
			Boys	Non-OB	201(56.8%)	18.3%	77(21.8%)	28.7%	4.825
				OB	45(12.7%)		31(8.8%)		(0.028)
			Girls	Non-OB	66(47.1%)	30.5%	24(17.1%)	48.3%	3.465
				OB	29(20.7%)		21(15%)		(0.063)

Supplementary Figure 1

De Finetti plot to assess the Hardy-Weinberg Equilibrium

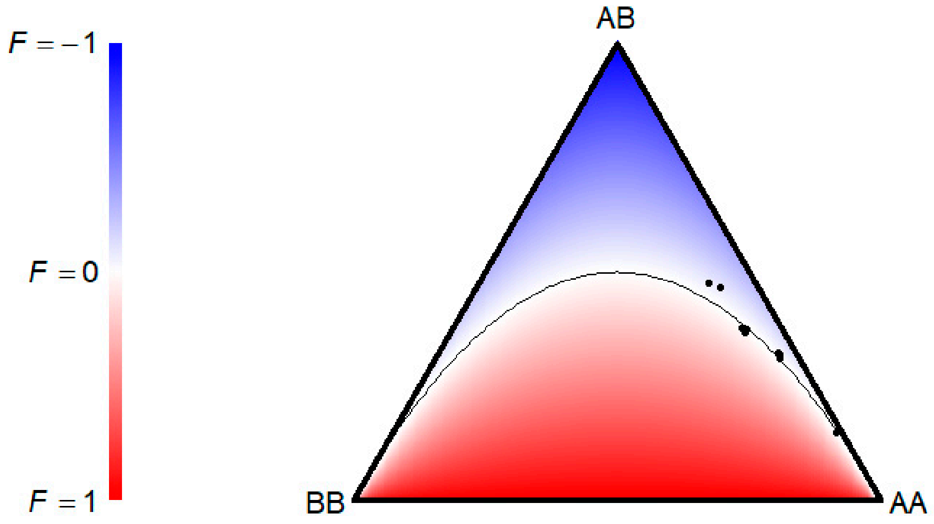

Supplement: Supplementary file 1 [file jpm-11-00091-s001.pdf]
